# The effects of temperature on elastic energy storage and release in a system with a dynamic mechanical advantage latch

**DOI:** 10.1242/jeb.245805

**Published:** 2023-10-12

**Authors:** Elizabeth Mendoza, Maya Martinez, Jeffrey P. Olberding, Emanuel Azizi

**Affiliations:** ^1^ Department of Ecology and Evolutionary Biology, University of California, Irvine, Irvine, CA 92697, USA; ^2^ Biomedical Engineering Department, California State University, Long Beach, CA 90840, USA; ^3^ Department of Biological Science, California State University, Fullerton, CA 92831, USA

**Keywords:** LaMSA, Elastic recoil, Jumping, Muscle, Temperature

## Abstract

Changes in temperature alter muscle kinetics and in turn affect whole-organism performance. Some organisms use the elastic recoil of biological springs, structures which are far less temperature sensitive, to power thermally robust movements. For jumping frogs, the use of elastic energy in tendons is facilitated through a geometric latching mechanism that operates through dynamic changes in the mechanical advantage (MA) of the hindlimb. Despite the well-documented use of elastic energy storage, frog jumping is a locomotor behavior that is significantly affected by changes in temperature. Here, we used an *in vitro* muscle preparation interacting in real time with an *in silico* model of a legged jumper to understand how changes in temperature affect the flow of energy in a system using a MA latch. We used the plantaris longus muscle–tendon unit (MTU) to power a virtual limb with changing MA and a mass being accelerated through a real-time feedback controller. We quantified the amount of energy stored in and recovered from elastic structures and the additional contribution of direct muscle work after unlatching. We found that temperature altered the duration of the energy loading and recovery phase of the *in vitro*/*in silico* experiments. We found that the early phase of loading was insensitive to changes in temperature. However, an increase in temperature did increase the rate of force development, which in turn allowed for increased energy storage in the second phase of loading. We also found that the contribution of direct muscle work after unlatching was substantial and increased significantly with temperature. Our results show that the thermal robustness achieved by an elastic mechanism depends strongly on the nature of the latch that mediates energy flow, and that the relative contribution of elastic and direct muscle energy likely shapes the thermal sensitivity of locomotor systems.

## Teaser

**Summary:** Thermal robustness, achieved through the use of elastic mechanisms, depends on the relative contribution of spring and muscle energy.

## INTRODUCTION

Many physiological processes are sensitive to changes in temperature (Bennett, 1984, 1985, 1990; Angilletta et al., 2002; James, 2013). This can pose a challenge to ectothermic organisms that rely on environmental conditions to regulate body temperature and physiological rates. In ectotherms, locomotor modes such as sprint speed, jump distance and swim speed have been shown to be sensitive to changes in temperature (Bennett, 1990; Hertz et al., 1982; Herrel et al., 2007; Navas, 1996; Peplowski and Marsh, 1997; Navas et al., 1999). These temperature-dependent changes in locomotor performance are linked to the strong thermal sensitivity of muscle contractile kinetics (e.g. shortening velocity, rate of force development, and power), and can have consequences for organism survival (Hertz et al., 1982; Putnam and Bennett, 1982; Bennett, 1984; Lutz and Rome, 1996; Peplowski and Marsh, 1997; James, 2013).

Temperature coefficients (*Q*_10_) are often used to indicate the rate of change of a variable when temperature is increased by 10**°**C. A *Q*_10_ equal to 1.0 indicates that the variable measured does not change much over a 10**°**C temperature range. A *Q*_10_ equal to 2.0 indicates that the variable measured increases by 2-fold over the range of temperatures measured. A *Q*_10_ equal to 0.5 indicates that the variable measured is halved over the range of temperatures measured (Bennett, 1984; Lutz and Rome, 1996; Peplowski and Marsh, 1997; James, 2013). Previous studies have used *Q*_10_ to understand how the thermal sensitivity of skeletal muscles informs the thermal sensitivity of locomotor performance (Else and Bennett, 1987; Swoap et al., 1993; Johnson et al., 1996; Peplowski and Marsh, 1997; Navas et al., 1999; Olberding and Deban, 2021).

Some ectothermic organisms use elastic recoil mechanisms to perform high-powered movements that are more robust to changes in temperature than what would be expected based on the *Q*_10_ of skeletal muscle (James, 2013; Olberding and Deban, 2021). Muscle contractile velocity, rate of force development or yank (Lin et al., 2019), and power show *Q*_10_ values that range between 1.5 and 3.0 (Putnam and Bennett, 1982; Bennett, 1984; Rome, 1990; Peplowski and Marsh, 1997) indicating that with a 10**°**C change in temperature there is a 2- to 3-fold increase in rate. In contrast, peak velocity, acceleration and power of elastically actuated tongue projection in chameleons shows *Q*_10_ values that range from 1.1 to 1.3 across 15–25**°**C, indicating that tongue projection is relatively insensitive to the change in temperature (Anderson and Deban, 2010). Thermal robustness through elastic recoil has also been observed in salamander tongue projection (Deban and Richardson, 2011; Scales et al., 2017; Deban et al., 2020) and in anuran tongue projection and ballistic mouth opening (Deban and Lappin, 2011; Sandusky and Deban, 2012), suggesting that elastic recoil mechanisms underlie thermally robust behaviors in some ectothermic organisms.

Elastic recoil mechanisms drive some of the fastest and most powerful movements ever recorded in biology (Ilton et al., 2018; Longo et al., 2019; Longo et al., 2021). In animals, these extremely fast and powerful behaviors are the result of finely tuned interactions between muscle, spring (e.g. tendon, aponeurosis, apodeme), latch and projectile (Ilton et al., 2018; Longo et al., 2019). In these systems, the muscle functions as the source of mechanical energy needed to acuate the system, the spring functions to temporarily store said energy, whereas the latch mediates the ability of the system to store and subsequently release mechanical energy loaded into the spring, often at a significantly higher rate than would be possible with direct muscle actuation. While the muscle and spring function are familiar and the structures involved well known, the latch can take many diverse forms. Here, we define a latch as any mechanism (sometimes referred to as a ‘catch’ in the literature) that provides a temporary force that resists motion in the ‘latched’ state while being able to be quickly removed or overcome to mediate the release of energy from a spring (Longo et al., 2019; Divi et al., 2020). Latches may be physical or anatomical structures where contact surfaces resists motion (Larabee et al., 2018). Latches may also result from geometric changes in a system where shifts in geometry can rapidly mediate a shift from storing energy in a spring to releasing that energy to actuate a projectile (Roberts and Marsh, 2003; Steinhardt et al., 2021).

Recent work examining the role of the latch in mediating energy flow has shown that the latch substantially influences elastic recoil performance (Ilton et al., 2018; Abbott et al., 2019; Divi et al., 2020). Previous modeling studies have shown that the amount of energy returned decreased with relatively slower latch removal velocities, indicating that the elastic recoil mechanism is sensitive to latch behavior (Ilton et al., 2018; Abbott et al., 2019; Divi et al., 2020; Acharya et al., 2021). However, it is not yet understood whether external factors such as temperature can affect latch performance and how changes to latch dynamics may allow for the contribution of direct muscle power to actuate the system.

Frog jumps are one of the most well-studied examples of elastically actuated movements (Marsh, 1994; Peplowski and Marsh, 1997; Roberts and Marsh, 2003; Azizi and Roberts, 2010, 2012; Astley et al., 2013; Astley and Roberts, 2014; Astley, 2016; Mendoza et al., 2020). Previous studies have shown a temporal decoupling of muscle contraction from joint movement as evidence of elastic energy storage at the ankle joint (Roberts and Marsh, 2003; Azizi and Roberts, 2010; Astley and Roberts, 2012). The elastic recoil mechanism in frog jumps is mediated by a dynamic mechanical advantage latch (a type of geometric latch), where the poor mechanical advantage of the hindlimb extensor muscles and the body's inertia resist hindlimb joint extension early in the jump and allow for energy storage. Once the hindlimb extensor muscles build sufficient force to overcome the latch, the hindlimb joints begin to extend, and the mechanical advantage of the extensor muscles improves rapidly to release energy (Roberts and Marsh, 2003; Olberding et al., 2019; Astley and Roberts, 2014). Results from previous studies suggest that the muscle may continue to shorten as the tendon recoils, providing some evidence that this latching mechanism allows for a combination of elastic and direct muscle energy to contribute to jumps (Roberts and Marsh, 2003; Azizi and Roberts, 2010; Astley and Roberts, 2012; Sutton et al., 2019).

Despite the use of elastic mechanisms, frog jumping performance remains temperature sensitive (Hirano and Rome, 1984; John-Alder et al., 1988; Whitehead et al., 1989; Rome, 1990; Marsh, 1994; Peplowski and Marsh, 1997; Navas et al., 1999; Olberding and Deban, 2021). For example, Hirano and Rome (1984) showed that jump power operated with *Q*_10_ of 2.67, jump velocity with a *Q*_10_ of 3.33, and jump distance with a *Q*_10_ of 1.58 between 15 and 25**°**C in the leopard frog (*Rana pipiens*). In this study, we investigated how the latching mechanics of the frog jump mechanism affect their ability to store and release energy across temperature. Here, we aimed to reveal the mechanism that causes frog jumps to be temperature dependent and to determine whether the observed thermal sensitivity is due to differences in energy storage or release. We used an *in vitro* muscle preparation coupled with an *in silico* model of a jumper because it enabled us to control muscle performance through direct stimulation, while allowing our muscles to interact with realistic movement dynamics. We hypothesized that the amount of energy stored (i.e. work) would not change with temperature as slowing muscle contractions would only increase the duration and not the amount of elastic energy storage. Additionally, muscle contractile rates are sensitive to temperature; thus, we hypothesized that the amount of energy released (i.e. work) would be temperature dependent because of the additional work that may be contributed by extensor muscles during elastic recoil and joint extension (Bennett, 1984, 1985; Rall and Woledge, 1990; Roberts and Marsh, 2003; Azizi and Roberts, 2010; Astley and Roberts, 2012).

## MATERIALS AND METHODS

### Animals

Six similarly sized (mean±s.e.m. mass 100.25±4.23 g) bullfrogs, *Rana catesbeiana* (Shaw 1802), were purchased from a herpetological vendor (Rana Ranch, Twin Falls, ID, USA). The animals were group housed in glass terraria, maintained at 20–21°C and were fed calcium-enriched crickets *ad libitum*. Animal husbandry and use were approved by the University of California, Irvine, Animal Care and Use Committee (protocol AUP 20-129).

### Muscle preparation

In this study, we followed methods outlined by Azizi and Roberts (2014) and Mendoza and Azizi (2021). Briefly, frogs were euthanized with a double-pithing protocol. Once death was confirmed, we measured the length of the tibiofibula segment with digital calipers. We isolated the sciatic nerve branch running along the right femur. Then, we exposed the plantaris longus muscle and implanted a sonomicrometry crystal between two muscle fascicles near the muscle origin. A second crystal was implanted ∼8 mm distal to the first and both were secured with 6-0 silk. After instrumentation, we isolated the muscle preparation from the body by detaching the muscle's distal tendon from the plantar fascia and isolating the knee joint (where the muscle originates). The instrumented muscle was secured to a fixed clamp at the knee joint and the distal tendon was threaded through a custom-made clamp. The clamp on the distal tendon was attached to a 50 N servomotor (Aurora Scientific Inc., Ontario, CA, USA). The sciatic nerve was threaded through a custom-made nerve cuff that was connected to a Grass S99D stimulator (Grass Technologies, Warwick, RI, USA), and was used to electrically stimulate the muscle. Finally, the muscle preparation was placed in a bath of circulating anuran Ringer's solution maintained at room temperature (e.g. 20°C) with a temperature controller circulator. The bath was continuously aerated with oxygen.

### Muscle property characterization

For each muscle, we determined optimal stimulation voltage by increasing the voltage of twitch contractions by 1 V increments until force stopped increasing with increasing voltage (9–11 V). Next, we determined optimal muscle length (*L*_o_) by characterizing the force–length curve using tetanic fixed-end contractions at variable lengths. *L*_o_ was defined as the length at which the muscle produced the highest peak force (*P*_o_). Muscle fascicle length changes were measured with sonomicrometry, and muscle force and muscle–tendon unit (MTU) length were measured with the servomotor. Tetanic stimulation consisted of 0.2 ms pulses at 65 pulses s^−1^ and durations of 500 ms. Once *P*_o_ was determined, muscles were set to the initial length that resulted in the highest force during our isometric tests for all experimental contractions. In most preparations, this value corresponded to an approximate initial length of ∼1.25 *L*_o_.

### Experimental set-up and mechanical advantage latch parameters

**Fig. 1.**
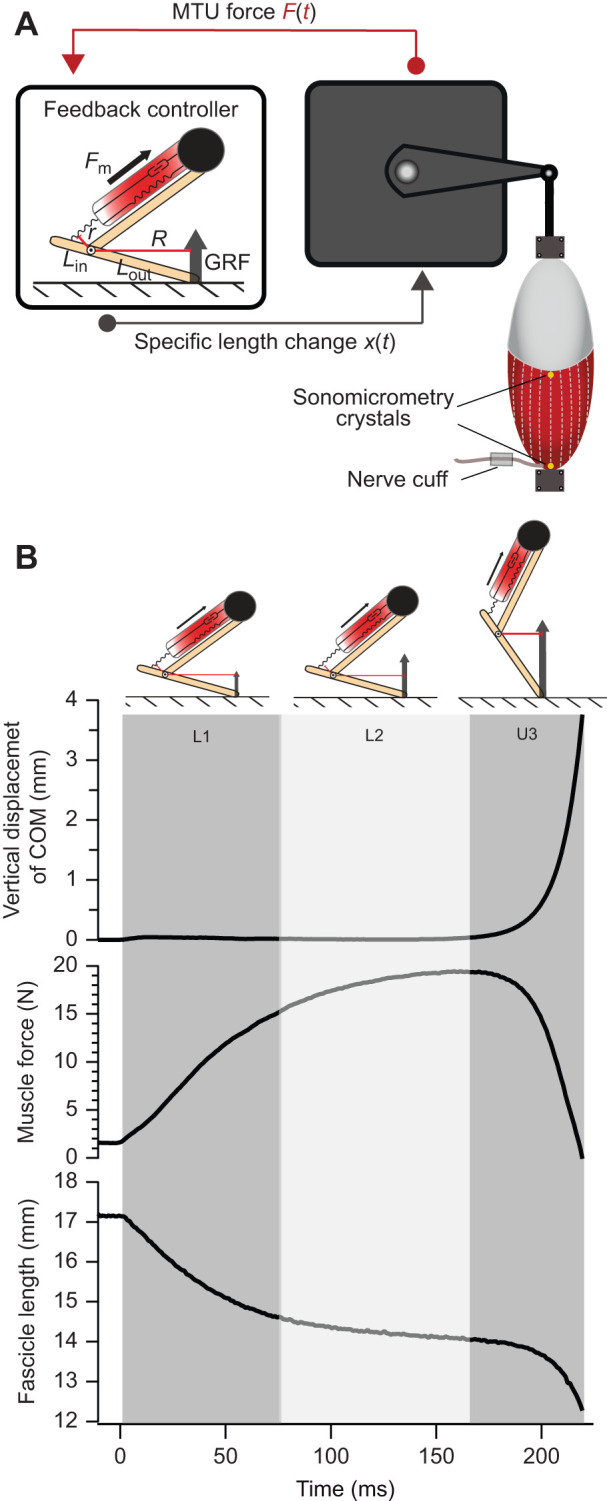
**Experimental set-up.** (A) An *in-vitro* muscle preparation was coupled with a real-time feedback controller programmed with a model of a virtual jumper from Olberding et al. (2019). To initiate the experiment, we set the muscle to optimal muscle length, *L*_o_, ran the model, and stimulated the muscle through a branch of the sciatic nerve. The servomotor read muscle force, and muscle force inputs were fed to the model by the servomotor. In response, the model outputted a calculated relative length change to the servomotor based on the muscle's mechanical advantage (MA). The joint began to extend when the muscle developed sufficient force to overcome the force threshold set by gravity acting through the mechanical advantage of the joint. The experiment ended when the joint extended to 180 deg or when force was equal to zero. Experiments were run at four temperatures: 10, 15, 20 and 25°C. MTU, muscle–tendon unit; *F*_m_, input muscle force; *r*, muscle moment arm; *R*, ground reaction force moment arm; *L*­_in_, in-lever length; *L*_out_, out-lever length; GRF, ground reaction force; *t*, time. (B) Example time series showing virtual center of mass (COM) position, muscle force and muscle fascicle length at 20°C. Changes in shading patterns indicate the transitions in the phases defined in our analysis: L1, first loading phase; L2, second loading phase; U3,unloading phase.

To investigate the effects of temperature on energy flow in a system with a dynamic mechanical advantage latch, we developed a novel *in vitro*/*in silico* muscle preparation. Specifically, our isolated muscle preparation interfaced with a BeagleBone Black computer with real-time feedback control that was programmed with Olberding et al.’s (2019) virtual jumper (Fig. 1A). Briefly, the modeled jumper consisted of two massless and frictionless segments that came together to form a first-class lever. In the model, a muscle is positioned parallel to the upper segment, and it applies input force (*F*_m_) at the end of the in-lever. Force applied by the muscle acts through the mechanical advantage of the joint and exerts a ground reaction force that accelerates a gravitational mass positioned at the end of the upper segment (see Olberding et al., 2019, for more details). For our experiments, we modified Olberding et al.’s (2019) jumper as follows. Segment length was set equal to the length of the right tibiofibula measured for each individual frog (*n*=6; mean±s.e.m. tibiofibular length 34.84±0.70 mm). We replaced the muscle in the model with an *in vitro* muscle preparation of the plantaris longus MTU and the force generated by the muscle *F*_m_ was fed into the model. The magnitude of the virtual projectile mass, *P*_mass_, was determined through the relationship of plantaris muscle mass to body mass using ratios from Marsh (1994) and Olson and Marsh (1998). As both plantaris MTUs were saved to be used in this and other experiments which required all the in-series tendon to be intact, muscle mass could not be accurately measured before the experiments. The projectile mass was therefore based on the body mass (*M*_b_) of each frog using:
(1)

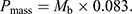
This calculation resulted in a ratio of plantaris mass to projectile mass that tracked natural variation in the ratio of hindlimb muscle mass to body mass in this species (0.16–0.21).

We calculated in-lever length, *L*_in_, based on the mechanical advantage, MA, required to overcome the gravitational load of the mass and inertia, given a peak force of 0.6*P*_o_ and a starting joint angle of 10 deg (see Table 1). The force level of 0.6*P*_o_ was selected based on previous muscle fascicle strain data during *in vivo* jumps showing that muscle fascicles shorten by about 10–15% prior to the movement of the center of mass. This value also allowed the muscles to reach this force level regardless of the slight effects of temperature on maximum isometric force (Olberding and Deban, 2017). We acknowledge that Olberding et al.’s (2019) model contains a calcaneus, and that frogs do not possess this anatomical structure. Again, the goal of the model was not to mimic a frog's anatomy or behavior, but rather to provide us with a controlled framework to compare patterns of energy flow through a system with a MA latch across a range of temperatures.

**Table 1.**
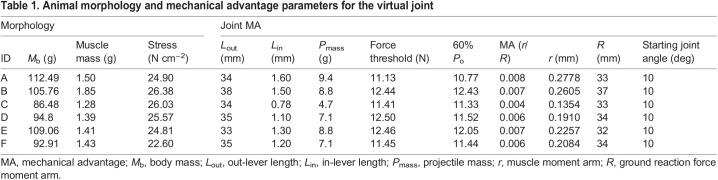
Animal morphology and mechanical advantage parameters for the virtual joint

### Temperature manipulations

After characterizing the force–length relationship, we ran the *in vitro*/*in silico* experiments across four temperature treatments: 10, 15, 20 and 25°C. In all experiments, the first set of tests were performed at 20°C followed by either sequence 10–15–25–20°C or 25–15–10–20°C. We ran the temperature experiments in this fashion to minimize the potential confounding interactions between muscle fatigue and temperature. The final tests at 20°C were compared with the initial tests at 20°C to make sure that the muscle had not dropped in its force capacity throughout the experiment. The difference in force during the initial and final tests at 20°C was used to examine the effect of treatment order on the muscle's overall performance. Treatment order did not have a significant effect on the trajectory of muscle force throughout the experiments (Student's *t*-test, *P*=0.31).

The temperature of the circulating Ringer's solution was manipulated with a temperature controller, and we monitored the solution's temperature with a temperature probe. Once the Ringer's solution reached treatment temperature, we waited 20 min to allow the muscle to reach the experimental temperature (Olberding and Deban, 2017). Then, the muscle was lengthened to *L*_o_, the model and the reactive feedback control loop were initiated, and the muscle was stimulated tetanically as described above. The details of the real-time feedback controller were previously described by Reynaga et al. (2019). Briefly, the servomotor registered the force generated by the muscle and broadcasted the analog signals in the ±10 V range. A peripheral 16-bit sampling analog-to-digital unit on the custom printed circuit board converted this signal to a digital force measurement, which was then passed via the serial peripheral interface bus to the BeagleBone, where it was smoothed with a software-implemented low-pass filter (Reynaga et al., 2019). The smoothed force and starting MA were used to calculate the displacement of the projectile's center of mass. The digital displacement value was then passed from the BeagleBone to a peripheral 16-bit multiplying digital-to-analog converter chip, where it was converted to an analog control signal. The analog control signal was sent to the servomotor, which controlled the position of the muscle lever (MTU length; Fig. 1A). This feedback loop continued until the muscle reached the force threshold. Once the muscle generated sufficient force to overcome the force threshold, the modeled joint began to extend and accelerate the mass. Experiments ended when the joint angle reached 180 deg or when force was equal to zero.

After experimentation, the muscle was detached from its origin at the knee joint and the distal free tendon was removed. Muscle mass, fascicle length and pennation angle were measured and used to calculate muscle stress using a known muscle density of 1.06 g cm^−2^ (Mendez and Keys, 1960). Muscle stress was calculated to ensure that the muscle preparations were of good quality (i.e. within physiological range, ∼20 N cm^−2^; Table 1) (Roberts et al., 2011; Mendoza and Azizi, 2021).

### Data processing

**Fig. 2.**
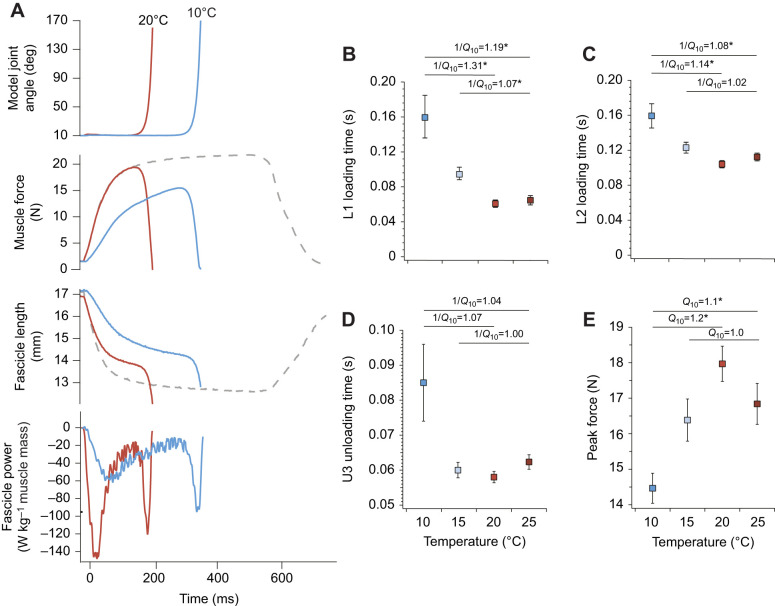
**Temperature effects on durations and muscle force.** (A) Example time series for experiments at 10 and 20°C showing model joint angle, muscle force, muscle fascicle length and muscle fascicle power. For comparison, an isometric tetanic fixed end contraction is shown as gray dashed lines. Note that if the muscle could not reach the force threshold, the contraction would proceed as an isometric tetanic fixed-end contraction. (B–E) Summary plots of (B) the duration of phase 1 of loading, (C) the duration of phase 2 of loading (D) the duration of unloading, and (E) peak muscle force plotted against temperature. Boxes are means and the error bars are s.e.m. Temperature coefficients significantly different from 1.0 are indicated with an asterisk.

All data were processed in Igor Pro software (Wavemetrics, Lake Oswego, OR, USA). To interrogate the role of a dynamic MA latch in mediating energy flow, we partitioned our data into three phases (Fig. 1B). The first loading phase (L1) was defined as the time from the beginning of muscle stimulation until a threshold force (0.6*P*_o_) was reached. During this phase, the latch was engaged, and the force required to accelerate the projectile had not been reached. The second loading phase (L2) was defined as the time from the muscle reaching the threshold force until the muscle reached its peak force. During this phase, the inertia of the projectile delays unlatching, providing additional time for energy to be loaded into elastic elements. Given that the model limb was not extending, the MTU was maintained at a constant length so any shortening of the muscle fascicles would result in an equal stretch of the elastic elements (Fig. 2A). Finally, the unloading phase (U3) was defined as the time at which the muscle reached peak force until the model took-off from the ground (Fig. 1B). In the unloading phase, the latch was removed, and this allowed the elastic elements to recoil while the muscle fascicles continued to do work during joint extension, simultaneously.

**Fig. 3.**
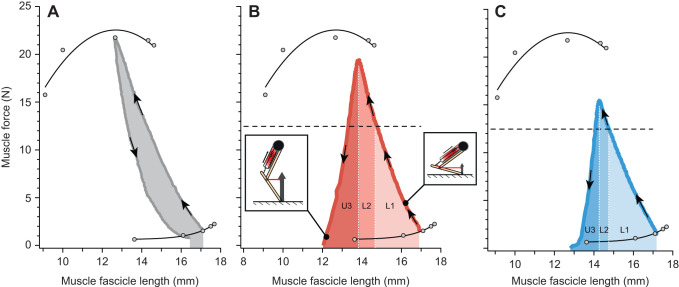
**Representative plots of mechanical work mapped onto the force–length curve for a muscle.** (A) Workloop of a tetanic fixed-end contraction. (B) A dynamic force–length plot for an experimental contraction at 20°C. (C) A dynamic force–length plot for an experimental contraction at 10°C. All three length trajectories began at a length of ∼17 mm with a passive force of ∼2 N and proceeded in the direction of the arrows. The changes in shading patterns in B and C correspond to the work done during the first phase of loading (L1), the second phase of loading (L2) and unloading (U3). The horizontal dashed line indicates the unlatching threshold for this muscle. For unlatching to initiate, the muscle needed to reach this force. Reaching the threshold force represents the transition from the first to the second phase of loading. During the second phase of loading, the muscle overshoots this threshold and continues to load energy into the tendon. The unloading muscle work represents energy that continues to be contributed by the muscle during tendon recoil (spring actuation phase).

The mechanical work (energy) associated with each of the three phases was calculated by plotting muscle fascicle length against muscle force during the loading phase and calculating the area under this curve (Fig. 3). We interrogated the unloading phase further by parsing out the relative contributions of tendon recoil work and direct muscle fascicle work (i.e. during joint extension). To do this, we measured total MTU work by plotting muscle force against MTU length during the unloading phase. The area under this curve was the total MTU work done during the unloading phase. To calculate tendon recoil work, we subtracted unloading muscle fascicle work from total MTU work. All measurements of work were converted to muscle mass-specific work by dividing by plantaris longus muscle mass.

We calculated tendon efficiency by taking the ratio of tendon recoil work to loading muscle fascicle work (L1 and L2). Additionally, we calculated the relative contributions of direct muscle fascicle work and tendon recoil work during joint extension, by taking the ratio of tendon recoil work to unloading muscle fascicle work. Lastly, we measured the duration of both the loading and unloading phase. We used this to calculate muscle fascicle power and tendon power.

### Analyses

All statistical analyses were performed in R (http://www.R-project.org/). We performed linear mixed models that included temperature as the continuous variable and individual as a random factor (Olberding and Deban, 2017; Olberding et al., 2018). For this analysis we used the function *lme* in the R package *nmle* (version 3.1). All dependent variables except for durations were log-transformed because their relationships were expected to be exponential with temperature. Dependent variables were analyzed separately across three temperature ranges: 10–20, 10–25 and 15–25°C. We used the partial regression coefficient of temperature to calculate temperature coefficients (*Q*_10_) using the equation:
(6)

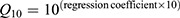


(Deban and Lappin, 2011; Deban and Richardson, 2011; Anderson and Deban, 2012; Scales et al., 2017; Olberding and Deban, 2017; Olberding et al., 2018). We reported *Q*_10_ of durations as inverse *Q*_10_ values (e.g. 1/*Q*_10_) to express them as rates (Deban and Lappin, 2011). The *P*-values for the regression coefficients for all tests were adjusted using the Benjamini–Hochberg procedure to control for false discovery (Benjamini and Hochberg, 1995). Temperature coefficients were significantly different from 1.0 if the *P*-value for the regression coefficients was less than the adjusted alpha.

## RESULTS

As expected, an increase in temperature resulted in a decrease in the duration of all phases of the experiments (Fig. 2A–C). Total loading time (L1 and L2) was longer than unloading time (U3). The duration of the first phase of loading (L1) decreased with increasing temperature, with the temperature coefficients differing significantly from 1.00 (Fig. 2B). Specifically, 10–20°C had a 1/*Q*_10_ of 1.31 (*P*=0.005), 15–25°C had a 1/*Q*_10_ of 1.07 (*P*=0.012), and 10–25°C had a 1/*Q*_10_ of 1.19 (*P*=0.006; Fig. 2B). The duration of the second phase of loading (L2) decreased with increasing temperature, with the temperature coefficients differing significantly from 1.00 for temperature ranges of 10–20°C and 10–25°C (Fig. 2C). Specifically, 10–20°C had a 1/*Q*_10_ of 1.14 (*P*=0.005), 15–25°C had a 1/*Q*_10_ of 1.02 (*P*=0.192), and 10–25°C had a 1/*Q*_10_ of 1.08 (*P*=0.015; Fig. 2C). Unloading time (U3) did not have temperature coefficients that were significantly different from 1.00 across all three temperature ranges. Specifically, the temperature coefficient at 10–20°C was 1/*Q*_10_=1.07 (*P*=0.035), 10–25°C was 1/*Q*_10_=1.04 (*P*=0.037), and 15–25°C was 1/*Q*_10_=1.00 (*P*=0.427; Fig. 2C).

We found that peak force increased with increasing temperature and peaked at 20°C (Fig. 2E). The *Q*_10_ for peak force across 10–20°C and 10–25°C was 1.2 and 1.1, respectively, both significantly different from 1.0 (*P*=0.00 and *P*=0.005, respectively). The *Q*_10_ for the temperature range 15–25°C was 1.00 (*P*=0.326; Fig. 2E).

**Fig. 4.**
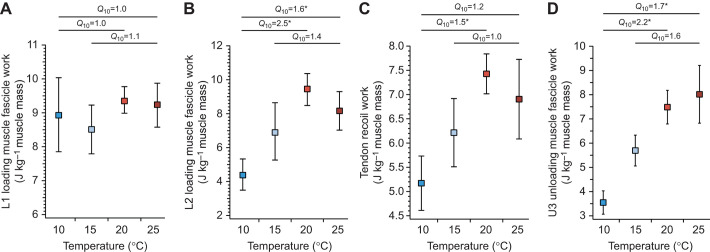
**Mass-specific work as a function of temperature.** (A) L1 loading muscle fascicle work, (B) L2 loading muscle fascicle work, (C) tendon recoil work and (D) U3 unloading muscle fascicle work plotted against temperature. Boxes are means and the error bars are s.e.m. Temperature coefficients significantly different from 1.0 are indicated with an asterisk.

Instantaneous force–length plots were used to quantify the flow of mechanical energy during *in vitro*/*in silico* contractions (Fig. 3). Muscle work generated during the first phase of loading (L1) did not vary with temperature (Fig. 3A,B and 4A). The *Q*_10_ for L1 muscle work did not significantly differ from 1.0 and was 1.0 for 10–20°C (*P*=0.765), 1.0 for 10–25°C (*P*=0.919), and 1.1 for 15–25°C (*P*=0.302; Fig. 4A). However, muscle work generated during the second phase of loading (L2) did vary with temperature over the 10–20°C and 10–25°C temperature ranges (Figs 3A,B and 4B). The *Q*_10_ for muscle work during L2 was 2.5 for 10–20°C (*P*=0.005), 1.6 for 10–25°C (*P*=0.014), and 1.4 for 15–25°C (*P*=0.296; Fig. 4B). Tendon recoil work increased with increasing temperature (Fig. 4C). The temperature range 10–20°C had a *Q*_10_ of 1.5, which was significantly different from 1.0 (*P*=0.003). The temperature range 10–25°C and 15–25°C had *Q*_10_ values equal to 1.2 (*P*=0.036) and 1.0 (*P*=0.316), respectively, which were not significantly different from 1.0 (Fig. 4C). Unloading muscle work increased significantly with increasing temperature (Figs 3B,C and 4D). The *Q*_10_ for unloading muscle work across all temperature ranges significantly differed from 1.0 and was 2.2 for 10–20°C (*P*=0.0004), 1.7 for 10–25°C (*P*=0.003), and 1.6 for 15–25°C (*P*=0.052; Fig. 4C).

**Fig. 5.**
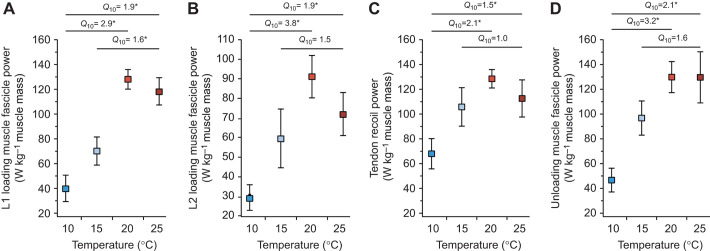
**Mass-specific power as a function of temperature.** (A) L1 loading muscle fascicle power, (B) L2 loading muscle fascicle power (C) tendon recoil power and (D) U3 unloading muscle fascicle power plotted against temperature. Boxes are means and the error bars are s.e.m. Temperature coefficients significantly different from 1.0 are indicated with an asterisk.

Muscle power during the first phase of loading (L1) increased with increasing temperature (Fig. 5A). The temperature coefficient was significantly different from 1.0 for the temperature range 10–20°C (*Q*_10_=2.9, *P*=0.002), 10–25°C (*Q*_10_=1.9, *P*=0.005; Fig. 5A), and 15–25°C (*Q*_10_=1.6, *P*=0.022; Fig. 5A). Muscle power during the second phase of loading (L2) increased with increasing temperature (Fig. 5B). The temperature coefficient was significantly different from 1.0 for the temperature range 10–20°C (*Q*_10_=3.8, *P*=0.001) and for the temperature range 10–25°C (*Q*_10_=1.9, *P*=0.005; Fig. 5A). The temperature coefficient was not significantly different from 1.0 for the temperature range 15–25°C (*Q*_10_=1.5, *P*=0.267; Fig. 5B). Tendon recoil power increased with increasing temperature (Fig. 5C). The temperature range 10–20°C had a *Q*_10_ of 2.1 (*P*=0.007) and the temperature range 10–25°C had a *Q*_10_ of 1.5 (*P*=0.016) and both were significantly different from 1.0 (Fig. 5C). The temperature range 15–25°C had a *Q*_10_ of 1.0 (*P*=0.985) and was not significantly different from 1.0 (Fig. 5C). Unloading muscle power increased with increasing temperature (Fig. 5D). The *Q*_10_ was significantly different from 1.0 for temperature range 10–20°C (*Q*_10_=3.2, *P*=0.0004) and 10–25°C (*Q*_10_=2.1, *P*=0.002; Fig. 5D). The *Q*_10_ was not significantly different from 1.0 for the temperature range 15–25°C (*Q*_10_=1.6, *P*=0.071; Fig. 5D).

**Fig. 6.**
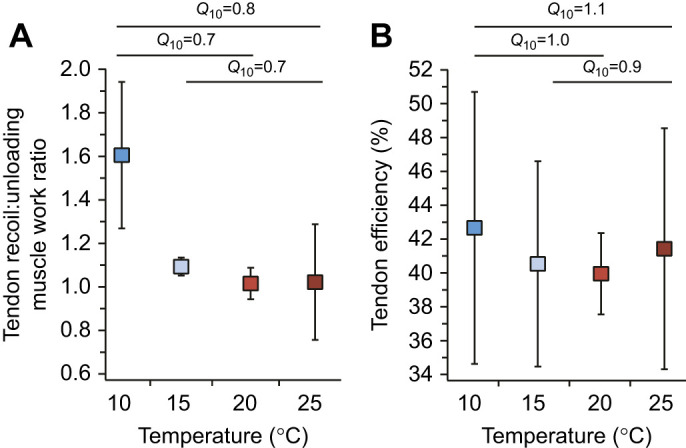
**Temperature effects on the ratio of tendon recoil energy to unloading muscle fascicle work ratio, and tendon efficiency.** (A) The energy associated with the recoil of the tendon makes up a larger of proportion of the total energy at lower temperatures whereas the muscle contributes more work at higher temperatures. (B) Tendon efficiency (percentage of energy returned) did not vary with temperature. Boxes are means and the error bars are s.e.m. Temperature coefficients significantly different from 1.0 are indicated with an asterisk.

The tendon recoil work to unloading muscle work ratio did not significantly change with increasing temperature (Fig. 6A). *Q*_10_ values for the temperature ranges 10–20°C (*Q*_10_=0.7; *P*=0.018), 10–25°C (*Q*_10_=0.8; *P*=0.120) and 15–25°C (*Q*_10_=0.7; *P*=0.217) were not significantly different from 1.0 (Fig. 6A). Efficiency did not change with temperature (Fig. 6B). Average efficiency was approximately (mean±s.e.m.) 44.69±4.47% across all temperature treatments. *Q*_10_ values for the temperature ranges 10–20°C (*Q*_10_=1.0; *P*=0.845), 10–25°C (*Q*_10_=1.1; *P*=0.581) and 15–25°C (*Q*_10_=0.9; *P*=0.739) were not significantly different from 1.0 (Fig. 6B).

## DISCUSSION

Thermal robustness achieved through spring actuation has been demonstrated in some ectothermic organisms (Anderson and Deban, 2010; Deban and Lappin, 2011; Deban and Richardson, 2011; Deban et al., 2020; Olberding and Deban, 2021). However, it appears that the degree of insensitivity to changes in temperature varies across systems. Jumping in frogs has been shown to be relatively more sensitive to changes in temperature than other systems (e.g. chameleons) despite documented use of spring actuation (Hirano and Rome, 1984; Azizi and Roberts, 2010; Astley and Roberts, 2012). This suggests that the specific mechanics of latch-mediated spring actuation (LaMSA) are critical for producing movements that are thermally robust. In this study, we investigated how the latching mechanics mediated energy flow in a jumper with a dynamic MA latch. We hypothesized that loading muscle fascicle work would not differ across temperature treatment because unlatching would not occur until the muscle reached the force threshold and lowering the temperature would only increase the time required to reach such a threshold. Moreover, we predicted that unloading muscle fascicle work would differ across temperature treatment because of temperature effects on muscle contractile rates (Bennett, 1984, 1985; Rall and Woledge, 1990). We did in fact find that the mechanical work the muscle generated during the first phase of loading was not affected by temperature (Fig. 4A). However, during the second phase, the loading time available for additional energy storage was determined by the inertia of the mass (projectile) and warm muscles generated force more quickly and better utilized this phase (Fig. 4B). This finding is consistent with theoretical analyses of relating spring dynamics and inertial loads, and highlights the significance of the rate of force generation (via changes in temperature or fiber type recruitment) in maximizing the time available for energy storage (Galantis and Woledge, 2003; Richards and Sawicki, 2012). We found that tendon recoil work showed temperature dependence that reflected the work pattern observed during the loading phase (Fig. 4B). Furthermore, we found that unloading muscle fascicle work showed strong temperature dependence which supported the hypothesis (Fig. 4D). Our results suggest that a dynamic mechanical advantage latch cannot fully decouple muscle contraction from joint motion, allowing for temperature effects to affect motion. Movements that are actuated by a combination of elastic recoil and direct muscle actuation will not display the thermal robustness observed in other LaMSA systems (Anderson and Deban, 2010; Deban and Lappin, 2011; Deban and Richardson, 2011; Deban et al., 2020; Olberding and Deban, 2021). Together, our results indicate that temporal decoupling of muscle contraction from movement is critical for thermally robust movements (e.g. chameleon tongue projection; Wainwright and Bennett, 1992; Anderson and Deban, 2010).

Previous studies have shown that the plantaris longus muscle continues to shorten during limb extension in frog jumps (Roberts and Marsh, 2003; Azizi and Roberts, 2010; Astley and Roberts, 2012), yet the relative importance of this contribution remained unknown because *in vivo* measurements of muscle force are difficult to acquire in frogs (but see Richards and Biewener, 2007; Moo et al., 2017). In our study, we found that muscle fascicle work during the unloading phase showed strong thermal dependence across all temperature ranges (Fig. 4D). We found that unloading muscle fascicle work operated with *Q*_10_ values of 2.2, 1.6 and 1.7 across the temperature ranges of 10–20°C, 15–25°C and 10–25°C, respectively. At the coldest treatment, the muscle was able to contribute on average about 3.7 J kg^−1^ muscle mass, while at the warmest temperature, the muscle contributed on average approximately 7.8 J kg^−1^ muscle mass (Fig. 4C). At warmer treatments, the amount of work contributed by the muscle fascicles during the unloading phase was on a par with that returned by tendon recoil (Figs 4C,D and 6A). Our results showed that the work contributions of muscle during the unloading phase are as important as those of tendon recoil and highlight the hybrid nature of this jump mechanism (Sutton et al., 2019; Olberding et al., 2019).

The unlatching mechanics affected the tendon recoil efficiency and introduced thermal sensitivity. In our experiments, we found that elastic recoil operated with an average efficiency of 44.69% across all temperatures (Fig. 6B). Efficiency is substantially lower than previous reports of approximately 90% efficiency in tendon when measured during relatively slow cyclical tensile conditions (e.g. Ker, 1981). This low efficiency is likely due to energy dissipation during unlatching. Abbott et al. (2019) modeled an elastic recoil system with an antagonist muscle as a latch and showed that unlatching velocity (i.e. muscle relaxation rate) was critical for determining whether power would be amplified or attenuated. They found that the fastest unlatching resulted in substantial power amplification and the slowest unlatching resulted in power attenuation. Furthermore, Divi et al. (2020) modeled latches with different latch removal velocities and showed that slower unlatching resulted in increased control of projectile launch at the cost of efficiency. While our study does not examine unlatching velocity, our estimates of efficiency suggest that there may be an emphasis on control of jump trajectory during actuation that may result in a tradeoff with efficiency. The studies mentioned above support our findings and collectively suggest that unlatching duration may serve as a form of control on output performance at the incurred cost of energy loss (Hyun et al., 2023). Furthermore, although integration of the two phases resulted in temperature-sensitive movements, it may be of importance for control. In the frog jumping mechanism, muscles loaded work into elastic elements while the latch was engaged and continued to do work during unlatching and limb extension. Continuous contribution of work by the muscle throughout the jump suggests that frogs may have the ability to control the performance and directionality of the jump after unlatching. In fact, the ability to impart neural control during take-off may be an important feature of systems using geometric latches (Sutton and Burrows, 2008) and may not be available to systems with faster latches (Sutton and Burrows, 2010). The ability to control a movement in real time after unlatching would likely not be possible in organisms where the muscle only contributes energy during energy storage, and where the latch temporally decouples energy storage from energy return (Roberts, 2019). Thus, latches like the anuran latch may result in reduced efficiency and robustness to environmental perturbations (e.g. temperature), but they afford the organism greater control of energy release and movement during the actuation phase.

Thermal robustness through elastic recoil is observed in chameleon and salamander tongue projection, and ballistic mouth opening in frogs and toads, but less so in frog or house cricket jumping (Hirano and Rome, 1984; Anderson and Deban, 2010; Deban and Richardson, 2011; Deban and Lappin, 2011; Scales et al., 2017; Olberding and Deban, 2021; Deban and Anderson, 2021). Deban and Anderson (2021) showed that jumping performance in house crickets was relatively more temperature sensitive than jumping in fleas and other insects despite use of elastic recoil mechanisms. The authors suggest that this could be due to additional muscle contributions during the takeoff phase, or dealing with high loads, which are known to result in temperature-dependent work outputs (Olberding and Deban, 2017). In our study, the required MA to overcome the latch was calculated with peak muscle force equal to 0.6*P*_o_ to reduce the load experienced by the muscle and fatigue; therefore, it is not likely that there was an interaction effect of high load and temperature. In our study, continuous contribution of work by the muscle fascicles and the unlatching mechanics resulted in the integration of the loading and unloading phases. This continuity between the two phases allowed for transmission of muscle thermal sensitivity into the energy stored and energy returned by elastic structures. The strongest temperature effects were observed during the unloading phase where the muscle contributed work during joint extension. Thus, while frog jump performance is relatively more insensitive to changes in temperature than underlying muscle contractile properties, frog jump performance is relatively more sensitive to changes in temperature than other thermally robust systems (e.g. chameleon tongue projection). Our work suggests that organisms that use elastic recoil mechanisms will perform temperature-independent movements only when the latch temporally decouples muscle contraction from energy release (i.e. no additional contribution by the muscle during actuation).

Among the many LaMSA systems studied to date, no system has shown a capacity to augment spring actuation with additional muscular work after the spring actuation has begun. While at first glance it would seem favorable for systems to augment mechanical energy output during the actuation phase by continuing to generate muscle work, this ‘hybrid’ actuation operates with some important constraints. It is likely that the duration of the take-off phase of a jumping frog is largely determined by the rate of energy release by tendons constraining the time available for muscle contributions. This would suggest that the power output of the muscle must remain high enough to contribute a substantial amount of mechanical work during a limited period of time. Therefore, it is reasonable to assume that some of the fastest LaMSA systems operating with a more idealized latch simply do not provide muscles enough time to contribute significantly once unlatching has occurred (Divi et al., 2020). This would suggest that in systems using hybrid actuation, maintenance of muscle power may be favored by natural selection whereas idealized systems may move to muscles specialized for high force production (Longo et al., 2019).

### Study limitations

In this study, we examined the effects of temperature on elastic energy storage and return in a system with a dynamic mechanical advantage latch. We found that continuous muscle contributions and the unlatching mechanics in this system allowed for integration of energy storage and energy release that resulted in temperature dependence. While the results here demonstrate the role of unlatching mechanics in mediating energy flow, there are limitations to our approach. First, we used an isolated muscle preparation coupled to a model of a jumper (Olberding et al., 2019) to understand the effects of temperature on an elastic recoil system with a dynamic MA latch. We used limb morphology and muscle mass to scale the jumper model to each frog. Our approach assumes that the muscle physiology of the plantaris longus is representative of hip and other hindlimb extensor muscles. The plantaris longus is a biarticulate muscle that flexes the knee and primarily extends the ankle (Olson and Marsh, 1998). It has bipennate architecture and in bullfrogs it has a large aponeurosis sheet wrapping around the muscle belly, which is distinct from some of the hip and hindlimb muscles. Yet, previous studies examining the properties of the plantaris longus muscle suggest that its contractile behavior may be consistent with other hindlimb muscles involved in jumping. For example, Astley (2016) and Mendoza et al. (2020) showed that the underlying muscle properties of the plantaris longus and semimembranosus (parallel fibered hip extensor and knee flexor) are similar across several species of frogs from diverse microhabitats. Additionally, Deban and Lappin (2011) and Olberding et al. (2018) showed that the contractile properties of muscles used in elastic recoil mechanisms are consistent with those of typical skeletal muscle. Furthermore, *in vivo* studies in jumping bullfrogs showed that several muscles spanning the hip and hindlimb have similar activation patterns to the plantaris longus during jump takeoff, suggesting that they have similar functional roles during a jump (Olson and Marsh, 1998). Another limitation to our approach is that we simplified neural control to supramaximal stimulation, despite significant modulation of activation during locomotion (Gillis and Biewener, 2000). Future studies are necessary to understand whether the patterns observed here reflect *in vivo* patterns in frog jumps at variable temperatures. The patterns observed in our study are confounded by the limitations outlined above, yet they provide testable hypotheses for future studies examining the temperature effects on elastic energy storage and return in frog jumping *in vivo*.

### Conclusions

Frog jumping performance is known to be relatively more sensitive to changes in temperature than other movements driven by elastic recoil. In this study, we investigated what aspects of the jump mechanism contributed to temperature sensitivity by examining the role of latching mechanics in mediating energy storage and release. We found that continuous muscle contributions and the mechanics of a dynamic mechanical advantage latch resulted in thermal sensitivity of energy storage and energy return. Furthermore, we found that hindlimb muscle plays a substantial role in actuating jumps in addition to the recoil of elastic structures. Finally, we propose that actuation through elastic recoil and direct muscle contributions results in some thermal sensitivity but allows for greater control and modulation of the jump in real time.

## References

[JEB245805C1] Abbott, E. M., Nezwek, T., Schmitt, D. and Sawicki, G. S. (2019). Hurry up and get out of the way! Exploring the limits of muscle-based latch systems for power amplification. *Integr. Comp. Biol.* 59, 1546-1558. 10.1093/icb/icz141 31418784

[JEB245805C2] Acharya, R., Challita, E. J., Ilton, M. and Saad Bhamla, M. (2021). The ultrafast snap of a finger is mediated by skin friction. *J. R. Soc. Interface* 18, 20210672. 10.1098/rsif.2021.0672 34784775 PMC8596009

[JEB245805C3] Anderson, C. V. and Deban, S. M. (2010). Ballistic tongue projection in chameleons maintains high performance at low temperature. *Proc. Natl Acad. Sci. USA* 107, 5495-5499. 10.1073/pnas.0910778107 20212130 PMC2851764

[JEB245805C4] Anderson, C. V. and Deban, S. M. (2012). Thermal effects on motor control and in vitro muscle dynamics of the ballistic tongue apparatus in chameleons. *J. Exp. Biol.* 215, 4345-4357. 23125336 10.1242/jeb.078881

[JEB245805C5] Angilletta, M. J., Jr, Niewiarowski, P. H. and Navas, C. A. (2002). The evolution of thermal physiology in ectotherms. *J. Therm. Biol.* 27, 249-268. 10.1016/S0306-4565(01)00094-8

[JEB245805C6] Astley, H. C. and Roberts, T. J. (2012). Evidence for a vertebrate catapult: elastic energy storage in the plantaris tendon during frog jumping. *Biol. Lett.* 8, 386-389. 10.1098/rsbl.2011.0982 22090204 PMC3367733

[JEB245805C7] Astley, H. C. (2016). The diversity and evolution of locomotor muscle properties in anurans. *J. Exp. Biol.* 219, 3163-3173. 10.1242/jeb.142315 27707867

[JEB245805C8] Astley, H. C. and Roberts, T. J. (2014). The mechanics of elastic loading and recoil in anuran jumping. *J. Exp. Biol.* 217, 4372-4378. 10.1242/jeb.110296 25520385

[JEB245805C9] Astley, H. C., Abbott, E. M., Azizi, E., Marsh, R. L. and Roberts, T. J. (2013). Chasing maximal performance: a cautionary tale from the celebrated jumping frogs of Calaveras County. *J. Exp. Biol.* 216, 3947-3953. 10.1242/jeb.090357 24133149

[JEB245805C10] Azizi, E. and Roberts, T. J. (2010). Muscle performance during frog jumping: influence of elasticity on muscle operating lengths. *Proc. R. Soc. B* 277, 1523-1530. 10.1098/rspb.2009.2051 PMC2871832 20106852

[JEB245805C11] Azizi, E. and Roberts, T. J. (2014). Geared up to stretch: pennate muscle behavior during active lengthening. *J. Exp. Biol.* 217, 376-381. 10.1242/jeb.094383 24477610 PMC4008126

[JEB245805C12] Bennett, A. F. (1984). Thermal dependence of muscle function. *Am. J. Physiol. Regul. Integr. Comp. Physiol.* 247, R217-R229. 10.1152/ajpregu.1984.247.2.R217 6380314

[JEB245805C13] Bennett, A. F. (1985). Temperature and muscle. *J. Exp. Biol.* 115, 333-344. 10.1242/jeb.115.1.333 3875678

[JEB245805C14] Benjamini, Y. and Hochberg, Y. (1995). Controlling the false discovery rate: a practical and powerful approach to multiple testing. *J. R. Stat. Soc. B (Methodological)* 57, 289-300. 10.1111/j.2517-6161.1995.tb02031.x

[JEB245805C15] Bennett, A. F. (1990). Thermal dependence of locomotor capacity. *Am. J. Physiol. Regul. Integr. Comp. Physiol.* 259, R253-R258. 10.1152/ajpregu.1990.259.2.R253 2201218

[JEB245805C16] Deban, S. M. and Anderson, C. V. (2021). Temperature effects on the jumping performance of house crickets. *J. Exp. Zool. A Ecol. Integr. Physiol.* 335, 659-667. 10.1002/jez.2510 34288598

[JEB245805C17] Deban, S. M. and Lappin, A. K. (2011). Thermal effects on the dynamics and motor control of ballistic prey capture in toads: maintaining high performance at low temperature. *J. Exp. Biol.* 214, 1333-1346. 10.1242/jeb.048405 21430211

[JEB245805C18] Deban, S. M. and Richardson, J. C. (2011). Cold-blooded snipers: thermal independence of ballistic tongue projection in the salamander *Hydromantes platycephalus*. *J. Exp. Zool. A Ecol. Genet. Physiol.* 315, 618-630. 10.1002/jez.708 21953778

[JEB245805C19] Deban, S. M., Scales, J. A., Bloom, S. V., Easterling, C. M., O'donnell, M. K. and Olberding, J. P. (2020). Evolution of a high-performance and functionally robust musculoskeletal system in salamanders. *Proc. Natl Acad. Sci. USA* 117, 10445-10454. 10.1073/pnas.1921807117 32341147 PMC7229748

[JEB245805C20] Divi, S., Ma, X., Ilton, M., St. Pierre, R., Eslami, B., Patek, S. N. and Bergbreiter, S. (2020). Latch-based control of energy output in spring actuated systems. *J. R. Soc. Interface* 17, 20200070. 10.1098/rsif.2020.0070 32693743 PMC7423419

[JEB245805C21] Else, P. L. and Bennett, A. F. (1987). The thermal dependence of locomotor performance and muscle contractile function in the salamander *Ambystoma tigrinum nebulosum*. *J. Exp. Biol.* 128, 219-233. 10.1242/jeb.128.1.219 3559463

[JEB245805C22] Galantis, A. and Woledge, R. C. (2003). The theoretical limits to the power output of a muscle–tendon complex with inertial and gravitational loads. *Proc. R. Soc. Lond. B Biol. Sci.* 270, 1493-1498. 10.1098/rspb.2003.2403 PMC1691403 12965015

[JEB245805C23] Gillis, G. B. and Biewener, A. A. (2000). Hindlimb extensor muscle function during jumping and swimming in the toad (*Bufo marinus*). *J. Exp. Biol.* 203, 3547-3563. 10.1242/jeb.203.23.3547 11060216

[JEB245805C24] Herrel, A., James, R. S. and Van Damme, R. (2007). Fight versus flight: physiological basis for temperature-dependent behavioral shifts in lizards. *J. Exp. Biol.* 210, 1762-1767. 10.1242/jeb.003426 17488939

[JEB245805C25] Hertz, P. E., Huey, R. B. and Nevo, E. (1982). Fight versus flight: body temperature influences defensive responses of lizards. *Anim. Behav.* 30, 676-679. 10.1016/S0003-3472(82)80137-1

[JEB245805C26] Hirano, M. and Rome, L. C. (1984). Jumping performance of frogs (*Rana pipiens*) as a function of muscle temperature. *J. Exp. Biol.* 108, 429-439. 10.1242/jeb.108.1.429

[JEB245805C27] Hyun, N. P., Olberding, J. P., De, A., Divi, S., Liang, X., Thomas, E., St. Pierre, R., Steinhardt, E., Jorge, J., Longo, S. J. et al. (2023). Spring and latch dynamics can act as control pathways in ultrafast systems. *Bioinspir. Biomim.* 18, 026002. 10.1088/1748-3190/acaa7c 36595244

[JEB245805C28] Ilton, M., Bhamla, M. S., Ma, X., Cox, S. M., Fitchett, L. L., Kim, Y., Koh, J. S., Krishnamurthy, D., Kuo, C. Y., Temel, F. Z. et al. (2018). The principles of cascading power limits in small, fast biological and engineered systems. *Science* 360, eaao1082. 10.1126/science.aao1082 29700237

[JEB245805C29] James, R. S. (2013). A review of the thermal sensitivity of the mechanics of vertebrate skeletal muscle. *J. Comp. Physiol. B* 183, 723-733. 10.1007/s00360-013-0748-1 23483325

[JEB245805C30] John-Alder, H. B., Morin, P. J. and Lawler, S. (1988). Thermal physiology, phenology, and distribution of tree frogs. *Am. Nat.* 132, 506-520. 10.1086/284868

[JEB245805C31] Johnson, T. P., Bennett, A. F. and Mclister, J. D. (1996). Thermal dependence and acclimation of fast start locomotion and its physiological basis in rainbow trout (*Oncorhynchus mykiss*). *Physiol. Zool.* 69, 276-292. 10.1086/physzool.69.2.30164184

[JEB245805C32] Ker, R. F. (1981). Dynamic tensile properties of the plantaris tendon of sheep (*Ovis aries*). *J. Exp. Biol.* 93, 283-302. 10.1242/jeb.93.1.283 7288354

[JEB245805C33] Larabee, F. J., Smith, A. A. and Suarez, A. V. (2018). Snap-jaw morphology is specialized for high-speed power amplification in the dracula ant, *Mystrium camillae*. *R. Soc. Open Sci.* 5, 181447. 10.1098/rsos.181447 30662749 PMC6304126

[JEB245805C34] Lin, D. C., Mcgowan, C. P., Blum, K. P. and Ting, L. H. (2019). Yank: the time derivative of force is an important biomechanical variable in sensorimotor systems. *J. Exp. Biol.* 222, jeb180414. 10.1242/jeb.180414 31515280 PMC6765171

[JEB245805C35] Longo, S. J., Cox, S. M., Azizi, E., Ilton, M., Olberding, J. P., St Pierre, R. and Patek, S. N. (2019). Beyond power amplification: latch-mediated spring actuation is an emerging framework for the study of diverse elastic systems. *J. Exp. Biol.* 222, jeb197889. 10.1242/jeb.197889 31399509

[JEB245805C36] Longo, S. J., Ray, W., Farley, G. M., Harrison, J., Jorge, J., Kaji, T., Palmer, A. R. and Patek, S. N. (2021). Snaps of a tiny amphipod push the boundary of ultrafast, repeatable movement. *Curr. Biol.* 31, R116-R117. 10.1016/j.cub.2020.12.025 33561405

[JEB245805C37] Lutz, G. J. and Rome, L. C. (1996). Muscle function during jumping in frogs. I. Sarcomere length change, EMG pattern, and jumping performance. *Am. J. Physiol. Cell Physiol.* 271, C563-C570. 10.1152/ajpcell.1996.271.2.C563 8769996

[JEB245805C38] Marsh, R. L. (1994). Jumping ability of anuran amphibians. *Adv. Vet. Sci. Comp. Med.* 38, 51-111. 7810380

[JEB245805C39] Mendez, J. and Keys, A. (1960). Density and composition of mammalian muscle. *Metabolism* 9, 184-188.

[JEB245805C40] Mendoza, E. (2023). Mechanical and physiological determinants of elastic energy storage. *PhD Dissertation*, University of California, Irvine.

[JEB245805C41] Mendoza, E. and Azizi, E. (2021). Tuned muscle and spring properties increase elastic energy storage. *J. Exp. Biol.* 224, jeb243180. 10.1242/jeb.243180 34821932 PMC10658917

[JEB245805C42] Mendoza, E., Azizi, E. and Moen, D. S. (2020). What explains vast differences in jumping power within a clade? Diversity, ecology and evolution of anuran jumping power. *Funct. Ecol.* 34, 1053-1063. 10.1111/1365-2435.13545

[JEB245805C43] Moo, E. K., Peterson, D. R., Leonard, T. R., Kaya, M. and Herzog, W. (2017). In vivo muscle force and muscle power during near-maximal frog jumps. *PLoS One* 12, e0173415. 10.1371/journal.pone.0173415 28282405 PMC5345813

[JEB245805C44] Navas, C. A. (1996). Metabolic physiology, locomotor performance, and thermal niche breadth in neotropical anurans. *Physiol. Zool.* 69, 1481-1501. 10.1086/physzool.69.6.30164271

[JEB245805C45] Navas, C. A., James, R. S., Wakeling, J. M., Kemp, K. M. and Johnston, I. A. (1999). An integrative study of the temperature dependence of whole animal and muscle performance during jumping and swimming in the frog *Rana temporaria*. *J. Comp. Physiol. B* 169, 588-596. 10.1007/s003600050259 10633564

[JEB245805C46] Olberding, J. P. and Deban, S. M. (2017). Effects of temperature and force requirements on muscle work and power output. *J. Exp. Biol.* 220, 2017-2025. 10.1242/jeb.153114 28314747

[JEB245805C48] Olberding, J. P. and Deban, S. M. (2021). Thermal robustness of biomechanical processes. *J. Exp. Biol.* 224, 1-10. 10.1093/icb/icz139 33397796

[JEB245805C47] Olberding, J. P., Scales, J. A. and Deban, S. M. (2018). Movements of vastly different performance have similar underlying muscle physiology. *J. Exp. Biol.* 221, jeb166900. 10.1242/jeb.166900 29212843

[JEB245805C49] Olberding, J. P., Deban, S. M., Rosario, M. V. and Azizi, E. (2019). Modeling the determinants of mechanical advantage during jumping: consequences for spring-and muscle-driven movement. *Integr. Comp. Biol.* 59, 1515-1524. 10.1093/icb/icz139 31397849

[JEB245805C50] Olson, J. M. and Marsh, R. L. (1998). Activation patterns and length changes in hindlimb muscles of the bullfrog *Rana catesbeiana* during jumping. *J. Exp. Biol.* 201, 2763-2777. 10.1242/jeb.201.19.2763 9732331

[JEB245805C51] Peplowski, M. M. and Marsh, R. L. (1997). Work and power output in the hindlimb muscles of Cuban tree frogs *Osteopilus septentrionalis* during jumping. *J. Exp. Biol.* 200, 2861-2870. 10.1242/jeb.200.22.2861 9344973

[JEB245805C52] Putnam, R. W. and Bennett, A. F. (1982). Thermal dependence of isometric contractile properties of lizard muscle. *J. Comp. Physiol. B* 147, 11-20. 10.1007/BF00689285

[JEB245805C53] Rall, J. A. and Woledge, R. C. (1990). Influence of temperature on mechanics and energetics of muscle contraction. *Am. J. Physiol. Regul. Integr. Comp. Physiol.* 259, R197-R203. 10.1152/ajpregu.1990.259.2.R197 2201213

[JEB245805C54] Reynaga, C. M., Eaton, C. E., Strong, G. A. and Azizi, E. (2019). Compliant substrates disrupt elastic energy storage in jumping tree frogs. *Integr. Comp. Biol.* 59, 1535-1545. 10.1093/icb/icz069 31141102

[JEB245805C55] Richards, C. T. and Biewener, A. A. (2007). Modulation of in vivo muscle power output during swimming in the African clawed frog (*Xenopus laevis*). *J. Exp. Biol.* 210, 3147-3159. 10.1242/jeb.005207 17766291

[JEB245805C56] Richards, C. T. and Sawicki, G. S. (2012). Elastic recoil can either amplify or attenuate muscle tendon power, depending on inertial vs. fluid dynamic loading. *J. Theor. Biol.* 313, 68-78. 22898554 10.1016/j.jtbi.2012.07.033

[JEB245805C57] Roberts, T. J. (2019). Some challenges of playing with power: does complex energy flow constrain neuromuscular performance? *Integr. Comp. Biol.* 59, 1619-1628. 10.1093/icb/icz108 31241134 PMC7968386

[JEB245805C58] Roberts, T. J. and Marsh, R. L. (2003). Probing the limits to muscle-powered accelerations: lessons from jumping bullfrogs. *J. Exp. Biol.* 206, 2567-2580. 10.1242/jeb.00452 12819264

[JEB245805C59] Roberts, T. J., Abbott, E. M. and Azizi, E. (2011). The weak link: do muscle properties determine locomotor performance in frogs? *Philos. Trans. R. Soc. B Biol. Sci.* 366, 1488-1495. 10.1098/rstb.2010.0326 PMC3130445 21502120

[JEB245805C60] Rome, L. C. (1990). Influence of temperature on muscle recruitment and muscle function in vivo. *Am. J. Physiol. Regul. Integr. Comp. Physiol.* 259, R210-R222. 10.1152/ajpregu.1990.259.2.R210 2201215

[JEB245805C61] Sandusky, P. E. and Deban, S. M. (2012). Temperature effects on the biomechanics of prey capture in the frog *Rana pipiens*. *J. Exp. Zool. A Ecol. Genet. Physiol.* 317, 595-607. 10.1002/jez.1751 22952141

[JEB245805C62] Scales, J. A., O'donnell, M. K. and Deban, S. M. (2017). Thermal sensitivity of motor control of muscle-powered versus elastically powered tongue projection in salamanders. *J. Exp. Biol.* 220, 938-951. 27956483 10.1242/jeb.145896

[JEB245805C63] Steinhardt, E., Hyun N-S, P., Koh, J. S., Freeburn, G., Rosen, M. H., Temel, F. Z., Patek, S. N. and Wood, R. J. (2021). A physical model of mantis shrimp for exploring the dynamics of ultra-fast systems. *Proc. Natl Acad. Sci. USA* 118, e2026833118. 10.1073/pnas.2026833118 34389671 PMC8379920

[JEB245805C64] Sutton, G. P. and Burrows, M. (2008). The mechanics of elevation control in locust jumping. *J. Comp. Physiol. A* 194, 557-563. 10.1007/s00359-008-0329-z 18373101

[JEB245805C65] Sutton, G. P. and Burrows, M. (2010). The mechanics of azimuth control in jumping by froghopper insects. *J. Exp. Biol.* 213, 1406-1416. 10.1242/jeb.036921 20400624

[JEB245805C66] Sutton, G. P., Mendoza, E., Azizi, E., Longo, S. J., Olberding, J. P., Ilton, M. and Patek, S. N. (2019). Why do large animals never actuate their jumps with latch-mediated springs? Because they can jump higher without them. *Integr. Comp. Biol.* 59, 1609-1618. 31399734 10.1093/icb/icz145 PMC6907395

[JEB245805C67] Swoap, S. J., Johnson, T. P., Josephson, R. K. and Bennett, A. F. (1993). Temperature, muscle power output and limitations on burst locomotor performance of the lizard *Dipsosaurus dorsalis*. *J. Exp. Biol.* 174, 185-197. 10.1242/jeb.174.1.185

[JEB245805C68] Wainwright, P. C. and Bennett, A. F. (1992). The mechanism of tongue projection in chameleons: I. Electromyographic tests of functional hypotheses. *J. Exp. Biol.* 168, 1-21. 10.1242/jeb.168.1.1

[JEB245805C69] Whitehead, P. J., Puckridge, J. T., Leigh, C. M. and Seymour, R. S. (1989). Effect of temperature on jump performance of the frog *Limnodynastes tasmaniensis*. *Physiol. Zool.* 62, 937-949. 10.1086/physzool.62.4.30157938

